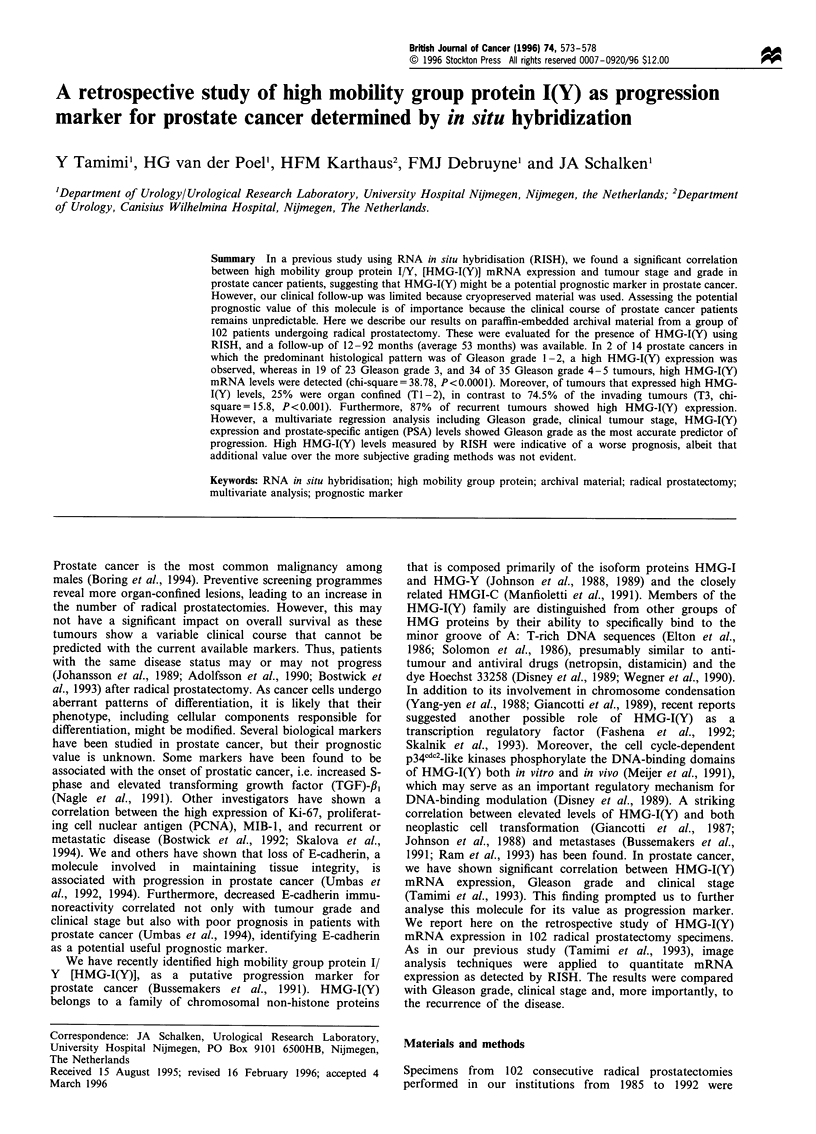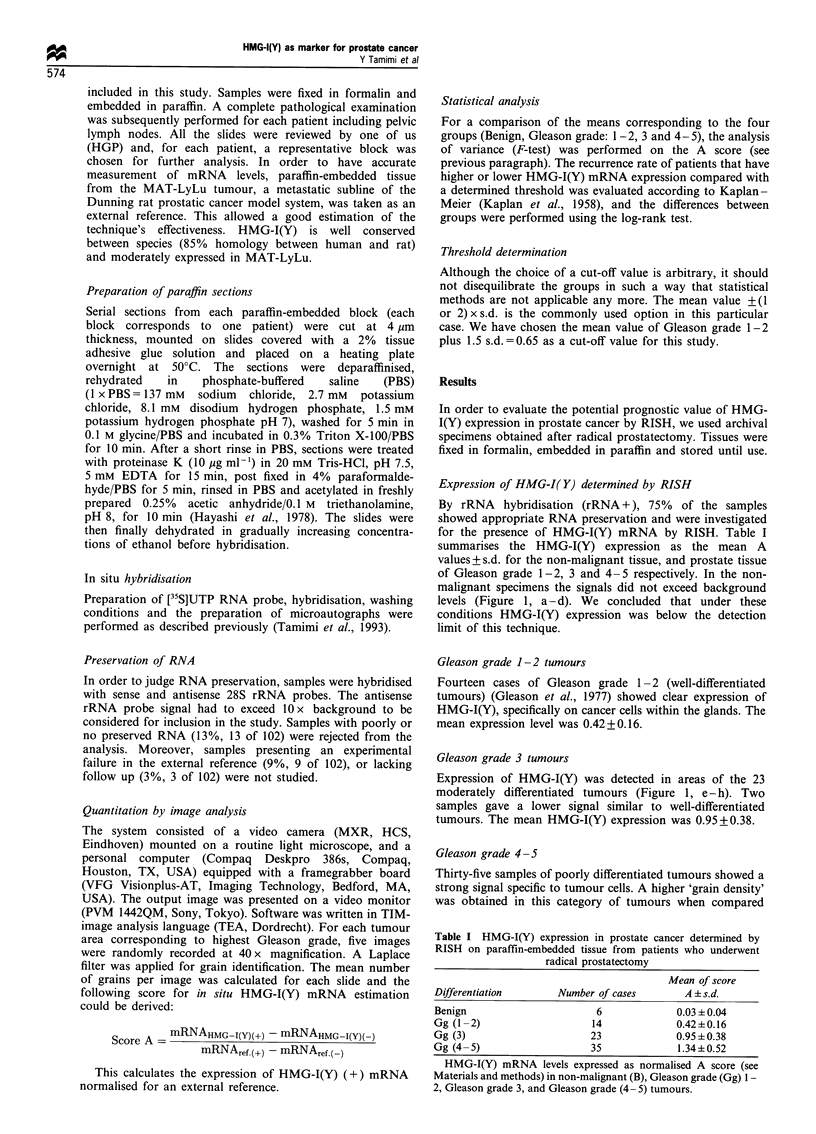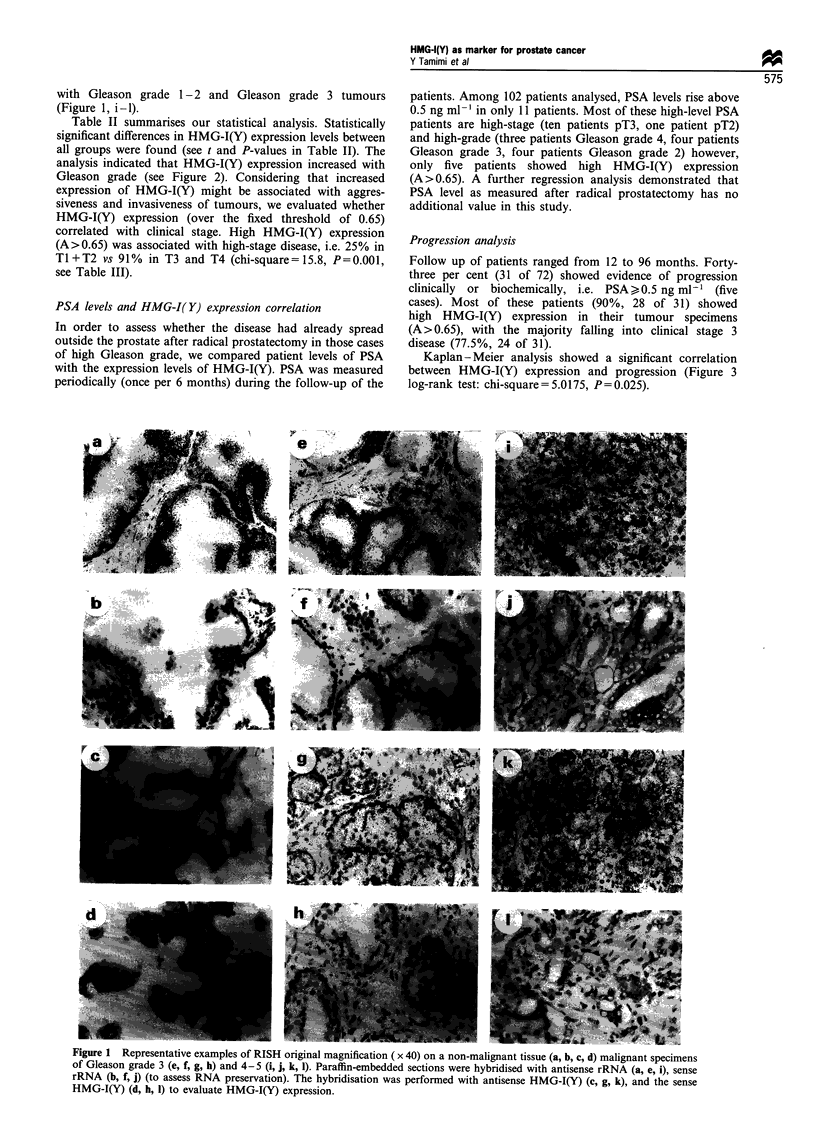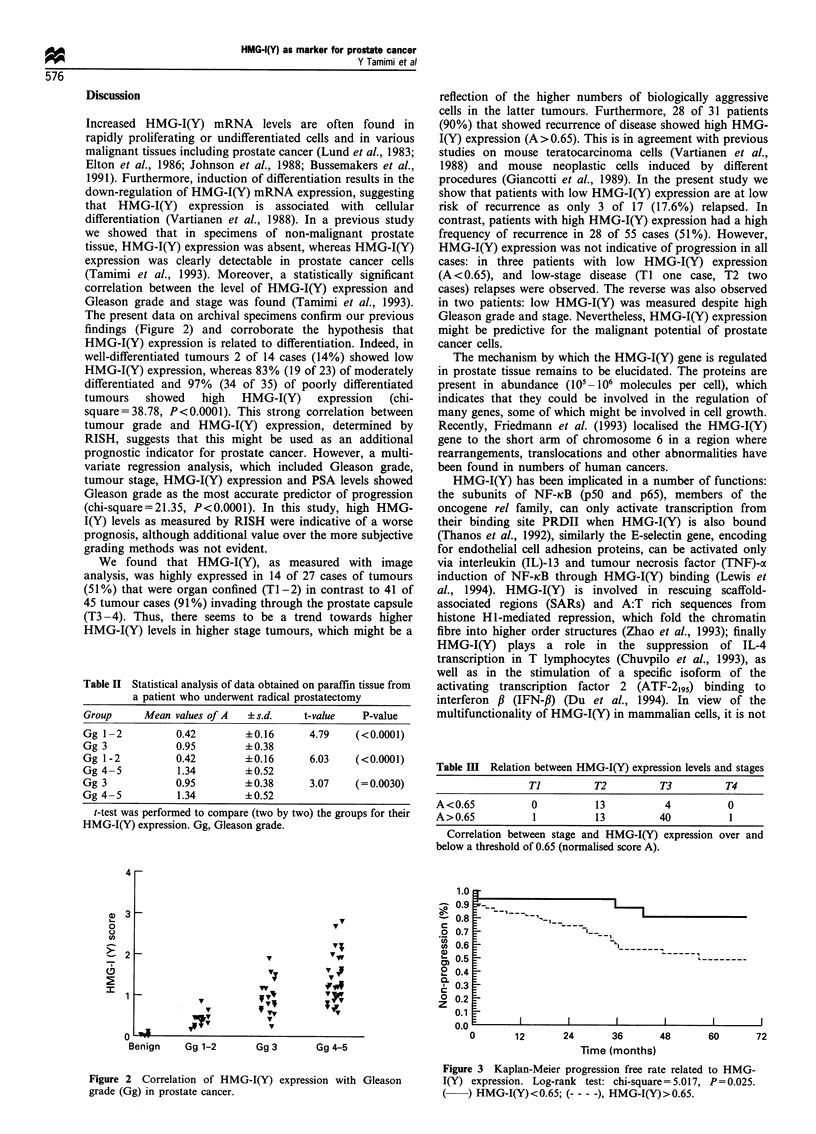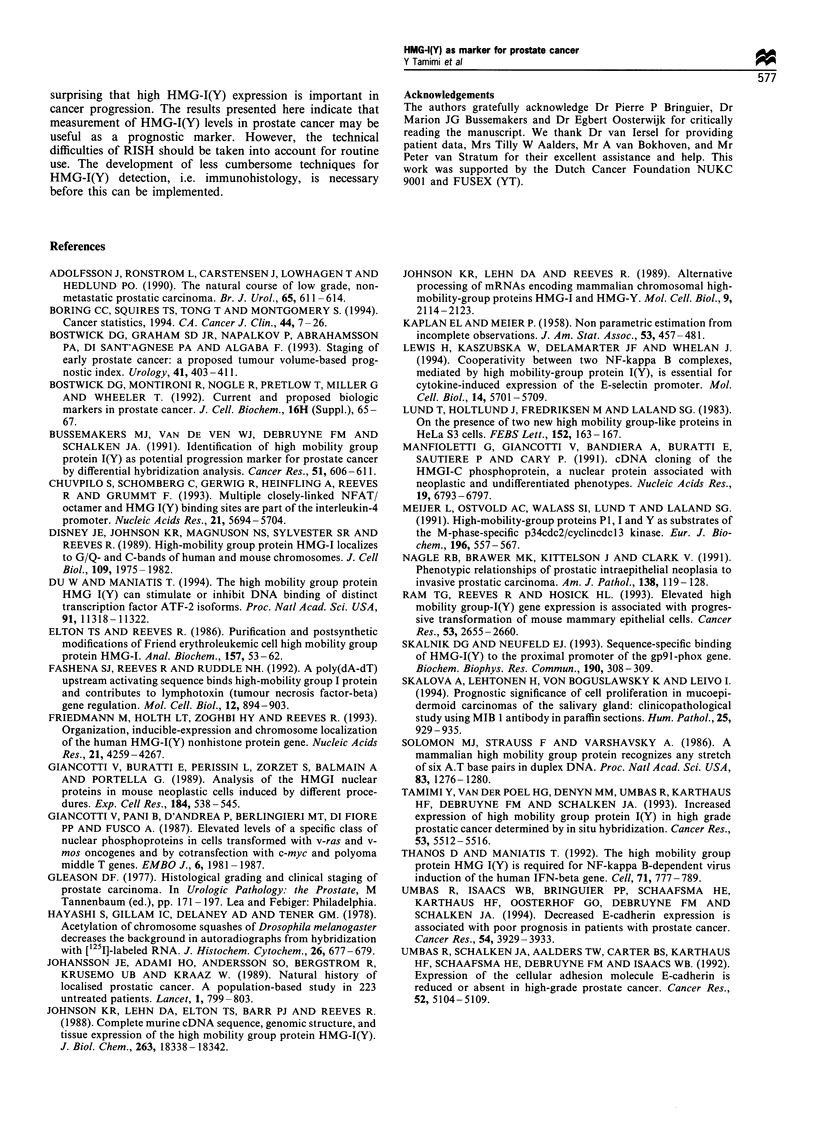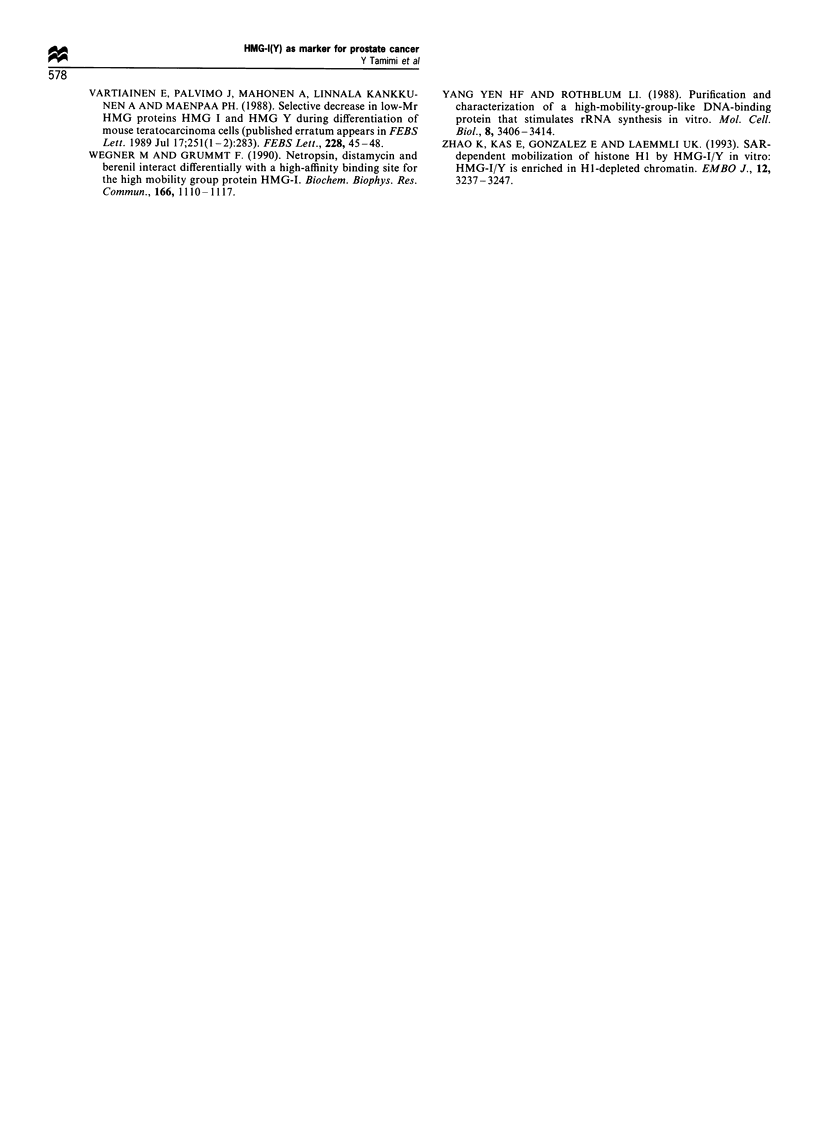# A retrospective study of high mobility group protein I(Y) as progression marker for prostate cancer determined by in situ hybridization.

**DOI:** 10.1038/bjc.1996.403

**Published:** 1996-08

**Authors:** Y. Tamimi, H. G. van der Poel, H. F. Karthaus, F. M. Debruyne, J. A. Schalken

**Affiliations:** Department of Urology/Urological Research Laboratory, University Hospital Nijmegen, The Netherlands.

## Abstract

**Images:**


British Journal of Cancer (1996) 74, 573-578
© 1996 Stockton Press All rights reserved 0007-0920/96 $12.00

### A retrospective study of high mobility group protein I(Y) as progression marker for prostate cancer determined by *in situ* hybridization

Y Tamimi[1], HG van der Poel[1], HFM Karthaus[2], FMJ Debruyne[1] and JA Schalken[1]

[1]*Department of Urology/Urological Research Laboratory, University Hospital Nijmegen, Nijmegen, the Netherlands;* [2]*Department of Urology, Canisius Wilhelmina Hospital, Nijmegen, The Netherlands.*

**Summary** In a previous study using RNA *in situ* hybridisation (RISH), we found a significant correlation between high mobility group protein I/Y, [HMG-I(Y)] mRNA expression and tumour stage and grade in prostate cancer patients, suggesting that HMG-I(Y) might be a potential prognostic marker in prostate cancer. However, our clinical follow-up was limited because cryopreserved material was used. Assessing the potential prognostic value of this molecule is of importance because the clinical course of prostate cancer patients remains unpredictable. Here we describe our results on paraffin-embedded archival material from a group of 102 patients undergoing radical prostatectomy. These were evaluated for the presence of HMG-I(Y) using RISH, and a follow-up of 12–92 months (average 53 months) was available. In 2 of 14 prostate cancers in which the predominant histological pattern was of Gleason grade 1–2, a high HMG-I(Y) expression was observed, whereas in 19 of 23 Gleason grade 3, and 34 of 35 Gleason grade 4–5 tumours, high HMG-I(Y) mRNA levels were detected (chi-square = 38.78, $P<0.0001$). Moreover, of tumours that expressed high HMG-I(Y) levels, 25% were organ confined (T1–2), in contrast to 74.5% of the invading tumours (T3, chi-square = 15.8, $P<0.001$). Furthermore, 87% of recurrent tumours showed high HMG-I(Y) expression. However, a multivariate regression analysis including Gleason grade, clinical tumour stage, HMG-I(Y) expression and prostate-specific antigen (PSA) levels showed Gleason grade as the most accurate predictor of progression. High HMG-I(Y) levels measured by RISH were indicative of a worse prognosis, albeit that additional value over the more subjective grading methods was not evident.

**Keywords:** RNA *in situ* hybridisation; high mobility group protein; archival material; radical prostatectomy; multivariate analysis; prognostic marker

Prostate cancer is the most common malignancy among males (Boring *et al.*, 1994). Preventive screening programmes reveal more organ-confined lesions, leading to an increase in the number of radical prostatectomies. However, this may not have a significant impact on overall survival as these tumours show a variable clinical course that cannot be predicted with the current available markers. Thus, patients with the same disease status may or may not progress (Johansson *et al.*, 1989; Adolfsson *et al.*, 1990; Bostwick *et al.*, 1993) after radical prostatectomy. As cancer cells undergo aberrant patterns of differentiation, it is likely that their phenotype, including cellular components responsible for differentiation, might be modified. Several biological markers have been studied in prostate cancer, but their prognostic value is unknown. Some markers have been found to be associated with the onset of prostatic cancer, i.e. increased S-phase and elevated transforming growth factor (TGF)-$\beta_1$ (Nagle *et al.*, 1991). Other investigators have shown a correlation between the high expression of Ki-67, proliferating cell nuclear antigen (PCNA), MIB-1, and recurrent or metastatic disease (Bostwick *et al.*, 1992; Skalova *et al.*, 1994). We and others have shown that loss of E-cadherin, a molecule involved in maintaining tissue integrity, is associated with progression in prostate cancer (Umbas *et al.*, 1992, 1994). Furthermore, decreased E-cadherin immunoreactivity correlated not only with tumour grade and clinical stage but also with poor prognosis in patients with prostate cancer (Umbas *et al.*, 1994), identifying E-cadherin as a potential useful prognostic marker.

We have recently identified high mobility group protein I/Y [HMG-I(Y)], as a putative progression marker for prostate cancer (Bussemakers *et al.*, 1991). HMG-I(Y) belongs to a family of chromosomal non-histone proteins that is composed primarily of the isoform proteins HMG-I and HMG-Y (Johnson *et al.*, 1988, 1989) and the closely related HMGI-C (Manfioletti *et al.*, 1991). Members of the HMG-I(Y) family are distinguished from other groups of HMG proteins by their ability to specifically bind to the minor groove of A: T-rich DNA sequences (Elton *et al.*, 1986; Solomon *et al.*, 1986), presumably similar to anti-tumour and antiviral drugs (netropsin, distamicin) and the dye Hoechst 33258 (Disney *et al.*, 1989; Wegner *et al.*, 1990). In addition to its involvement in chromosome condensation (Yang-yen *et al.*, 1988; Giancotti *et al.*, 1989), recent reports suggested another possible role of HMG-I(Y) as a transcription regulatory factor (Fashena *et al.*, 1992; Skalnik *et al.*, 1993). Moreover, the cell cycle-dependent $p34^{cdc2}$-like kinases phosphorylate the DNA-binding domains of HMG-I(Y) both *in vitro* and *in vivo* (Meijer *et al.*, 1991), which may serve as an important regulatory mechanism for DNA-binding modulation (Disney *et al.*, 1989). A striking correlation between elevated levels of HMG-I(Y) and both neoplastic cell transformation (Giancotti *et al.*, 1987; Johnson *et al.*, 1988) and metastases (Bussemakers *et al.*, 1991; Ram *et al.*, 1993) has been found. In prostate cancer, we have shown significant correlation between HMG-I(Y) mRNA expression, Gleason grade and clinical stage (Tamimi *et al.*, 1993). This finding prompted us to further analyse this molecule for its value as progression marker. We report here on the retrospective study of HMG-I(Y) mRNA expression in 102 radical prostatectomy specimens. As in our previous study (Tamimi *et al.*, 1993), image analysis techniques were applied to quantitate mRNA expression as detected by RISH. The results were compared with Gleason grade, clinical stage and, more importantly, to the recurrence of the disease.

### Materials and methods

Specimens from 102 consecutive radical prostatectomies performed in our institutions from 1985 to 1992 were

Correspondence: JA Schalken, Urological Research Laboratory, University Hospital Nijmegen, PO Box 9101 6500HB, Nijmegen, The Netherlands
Received 15 August 1995; revised 16 February 1996; accepted 4 March 1996

included in this study. Samples were fixed in formalin and embedded in paraffin. A complete pathological examination was subsequently performed for each patient including pelvic lymph nodes. All the slides were reviewed by one of us (HGP) and, for each patient, a representative block was chosen for further analysis. In order to have accurate measurement of mRNA levels, paraffin-embedded tissue from the MAT-LyLu tumour, a metastatic subline of the Dunning rat prostatic cancer model system, was taken as an external reference. This allowed a good estimation of the technique's effectiveness. HMG-I(Y) is well conserved between species (85% homology between human and rat) and moderately expressed in MAT-LyLu.

*Preparation of paraffin sections*

Serial sections from each paraffin-embedded block (each block corresponds to one patient) were cut at 4 $\mu$m thickness, mounted on slides covered with a 2% tissue adhesive glue solution and placed on a heating plate overnight at 50°C. The sections were deparaffinised, rehydrated in phosphate-buffered saline (PBS) (1×PBS = 137 mM sodium chloride, 2.7 mM potassium chloride, 8.1 mM disodium hydrogen phosphate, 1.5 mM potassium hydrogen phosphate pH 7), washed for 5 min in 0.1 M glycine/PBS and incubated in 0.3% Triton X-100/PBS for 10 min. After a short rinse in PBS, sections were treated with proteinase K (10 $\mu$g $ml^{-1}$) in 20 mM Tris-HCl, pH 7.5, 5 mM EDTA for 15 min, post fixed in 4% paraformaldehyde/PBS for 5 min, rinsed in PBS and acetylated in freshly prepared 0.25% acetic anhydride/0.1 M triethanolamine, pH 8, for 10 min (Hayashi *et al.*, 1978). The slides were then finally dehydrated in gradually increasing concentrations of ethanol before hybridisation.

In situ *hybridisation*

Preparation of [$^{35}$S]UTP RNA probe, hybridisation, washing conditions and the preparation of microautographs were performed as described previously (Tamimi *et al.*, 1993).

*Preservation of RNA*

In order to judge RNA preservation, samples were hybridised with sense and antisense 28S rRNA probes. The antisense rRNA probe signal had to exceed 10× background to be considered for inclusion in the study. Samples with poorly or no preserved RNA (13%, 13 of 102) were rejected from the analysis. Moreover, samples presenting an experimental failure in the external reference (9%, 9 of 102), or lacking follow up (3%, 3 of 102) were not studied.

*Quantitation by image analysis*

The system consisted of a video camera (MXR, HCS, Eindhoven) mounted on a routine light microscope, and a personal computer (Compaq Deskpro 386s, Compaq, Houston, TX, USA) equipped with a framegrabber board (VFG Visionplus-AT, Imaging Technology, Bedford, MA, USA). The output image was presented on a video monitor (PVM 1442QM, Sony, Tokyo). Software was written in TIM-image analysis language (TEA, Dordrecht). For each tumour area corresponding to highest Gleason grade, five images were randomly recorded at 40× magnification. A Laplace filter was applied for grain identification. The mean number of grains per image was calculated for each slide and the following score for *in situ* HMG-I(Y) mRNA estimation could be derived:

$$\text{Score A} = \frac{\text{mRNA}_{\text{HMG-I(Y)}(+)} - \text{mRNA}_{\text{HMG-I(Y)}(-)}}{\text{mRNA}_{\text{ref.}(+)} - \text{mRNA}_{\text{ref.}(-)}}$$

This calculates the expression of HMG-I(Y) (+) mRNA normalised for an external reference.

*Statistical analysis*

For a comparison of the means corresponding to the four groups (Benign, Gleason grade: 1–2, 3 and 4–5), the analysis of variance (*F*-test) was performed on the A score (see previous paragraph). The recurrence rate of patients that have higher or lower HMG-I(Y) mRNA expression compared with a determined threshold was evaluated according to Kaplan–Meier (Kaplan *et al.*, 1958), and the differences between groups were performed using the log-rank test.

*Threshold determination*

Although the choice of a cut-off value is arbitrary, it should not disequilibrate the groups in such a way that statistical methods are not applicable any more. The mean value ±(1 or 2)×s.d. is the commonly used option in this particular case. We have chosen the mean value of Gleason grade 1–2 plus 1.5 s.d. = 0.65 as a cut-off value for this study.

### Results

In order to evaluate the potential prognostic value of HMG-I(Y) expression in prostate cancer by RISH, we used archival specimens obtained after radical prostatectomy. Tissues were fixed in formalin, embedded in paraffin and stored until use.

*Expression of HMG-I(Y) determined by RISH*

By rRNA hybridisation (rRNA+), 75% of the samples showed appropriate RNA preservation and were investigated for the presence of HMG-I(Y) mRNA by RISH. Table I summarises the HMG-I(Y) expression as the mean A values ± s.d. for the non-malignant tissue, and prostate tissue of Gleason grade 1–2, 3 and 4–5 respectively. In the non-malignant specimens the signals did not exceed background levels (Figure 1, a–d). We concluded that under these conditions HMG-I(Y) expression was below the detection limit of this technique.

*Gleason grade 1–2 tumours*

Fourteen cases of Gleason grade 1–2 (well-differentiated tumours) (Gleason *et al.*, 1977) showed clear expression of HMG-I(Y), specifically on cancer cells within the glands. The mean expression level was 0.42±0.16.

*Gleason grade 3 tumours*

Expression of HMG-I(Y) was detected in areas of the 23 moderately differentiated tumours (Figure 1, e–h). Two samples gave a lower signal similar to well-differentiated tumours. The mean HMG-I(Y) expression was 0.95±0.38.

*Gleason grade 4–5*

Thirty-five samples of poorly differentiated tumours showed a strong signal specific to tumour cells. A higher 'grain density' was obtained in this category of tumours when compared

**Table I** HMG-I(Y) expression in prostate cancer determined by RISH on paraffin-embedded tissue from patients who underwent radical prostatectomy

| *Differentiation* | *Number of cases* | *Mean of score A ± s.d.* |
|---|---|---|
| Benign | 6 | 0.03±0.04 |
| Gg (1–2) | 14 | 0.42±0.16 |
| Gg (3) | 23 | 0.95±0.38 |
| Gg (4–5) | 35 | 1.34±0.52 |

HMG-I(Y) mRNA levels expressed as normalised A score (see Materials and methods) in non-malignant (B), Gleason grade (Gg) 1–2, Gleason grade 3, and Gleason grade (4–5) tumours.

with Gleason grade 1–2 and Gleason grade 3 tumours (Figure 1, i–l).

Table II summarises our statistical analysis. Statistically significant differences in HMG-I(Y) expression levels between all groups were found (see $t$ and $P$-values in Table II). The analysis indicated that HMG-I(Y) expression increased with Gleason grade (see Figure 2). Considering that increased expression of HMG-I(Y) might be associated with aggressiveness and invasiveness of tumours, we evaluated whether HMG-I(Y) expression (over the fixed threshold of 0.65) correlated with clinical stage. High HMG-I(Y) expression ($A>0.65$) was associated with high-stage disease, i.e. 25% in T1+T2 *vs* 91% in T3 and T4 (chi-square=15.8, $P=0.001$, see Table III).

### *PSA levels and HMG-I(Y) expression correlation*

In order to assess whether the disease had already spread outside the prostate after radical prostatectomy in those cases of high Gleason grade, we compared patient levels of PSA with the expression levels of HMG-I(Y). PSA was measured periodically (once per 6 months) during the follow-up of the patients. Among 102 patients analysed, PSA levels rise above 0.5 ng $ml^{-1}$ in only 11 patients. Most of these high-level PSA patients are high-stage (ten patients pT3, one patient pT2) and high-grade (three patients Gleason grade 4, four patients Gleason grade 3, four patients Gleason grade 2) however, only five patients showed high HMG-I(Y) expression ($A>0.65$). A further regression analysis demonstrated that PSA level as measured after radical prostatectomy has no additional value in this study.

### *Progression analysis*

Follow up of patients ranged from 12 to 96 months. Forty-three per cent (31 of 72) showed evidence of progression clinically or biochemically, i.e. PSA$\geqslant$0.5 ng $ml^{-1}$ (five cases). Most of these patients (90%, 28 of 31) showed high HMG-I(Y) expression in their tumour specimens ($A>0.65$), with the majority falling into clinical stage 3 disease (77.5%, 24 of 31).

Kaplan–Meier analysis showed a significant correlation between HMG-I(Y) expression and progression (Figure 3 log-rank test: chi-square=5.0175, $P=0.025$).

**Figure 1** Representative examples of RISH original magnification (×40) on a non-malignant tissue (**a, b, c, d**) malignant specimens of Gleason grade 3 (**e, f, g, h**) and 4–5 (**i, j, k, l**). Paraffin-embedded sections were hybridised with antisense rRNA (**a, e, i**), sense rRNA (**b, f, j**) (to assess RNA preservation). The hybridisation was performed with antisense HMG-I(Y) (**c, g, k**), and the sense HMG-I(Y) (**d, h, l**) to evaluate HMG-I(Y) expression.

### Discussion

Increased HMG-I(Y) mRNA levels are often found in rapidly proliferating or undifferentiated cells and in various malignant tissues including prostate cancer (Lund *et al.*, 1983; Elton *et al.*, 1986; Johnson *et al.*, 1988; Bussemakers *et al.*, 1991). Furthermore, induction of differentiation results in the down-regulation of HMG-I(Y) mRNA expression, suggesting that HMG-I(Y) expression is associated with cellular differentiation (Vartianen *et al.*, 1988). In a previous study we showed that in specimens of non-malignant prostate tissue, HMG-I(Y) expression was absent, whereas HMG-I(Y) expression was clearly detectable in prostate cancer cells (Tamimi *et al.*, 1993). Moreover, a statistically significant correlation between the level of HMG-I(Y) expression and Gleason grade and stage was found (Tamimi *et al.*, 1993). The present data on archival specimens confirm our previous findings (Figure 2) and corroborate the hypothesis that HMG-I(Y) expression is related to differentiation. Indeed, in well-differentiated tumours 2 of 14 cases (14%) showed low HMG-I(Y) expression, whereas 83% (19 of 23) of moderately differentiated and 97% (34 of 35) of poorly differentiated tumours showed high HMG-I(Y) expression (chi-square = 38.78, $P<0.0001$). This strong correlation between tumour grade and HMG-I(Y) expression, determined by RISH, suggests that this might be used as an additional prognostic indicator for prostate cancer. However, a multivariate regression analysis, which included Gleason grade, tumour stage, HMG-I(Y) expression and PSA levels showed Gleason grade as the most accurate predictor of progression (chi-square = 21.35, $P<0.0001$). In this study, high HMG-I(Y) levels as measured by RISH were indicative of a worse prognosis, although additional value over the more subjective grading methods was not evident.

We found that HMG-I(Y), as measured with image analysis, was highly expressed in 14 of 27 cases of tumours (51%) that were organ confined (T1–2) in contrast to 41 of 45 tumour cases (91%) invading through the prostate capsule (T3–4). Thus, there seems to be a trend towards higher HMG-I(Y) levels in higher stage tumours, which might be a reflection of the higher numbers of biologically aggressive cells in the latter tumours. Furthermore, 28 of 31 patients (90%) that showed recurrence of disease showed high HMG-I(Y) expression ($A>0.65$). This is in agreement with previous studies on mouse teratocarcinoma cells (Vartianen *et al.*, 1988) and mouse neoplastic cells induced by different procedures (Giancotti *et al.*, 1989). In the present study we show that patients with low HMG-I(Y) expression are at low risk of recurrence as only 3 of 17 (17.6%) relapsed. In contrast, patients with high HMG-I(Y) expression had a high frequency of recurrence in 28 of 55 cases (51%). However, HMG-I(Y) expression was not indicative of progression in all cases: in three patients with low HMG-I(Y) expression ($A<0.65$), and low-stage disease (T1 one case, T2 two cases) relapses were observed. The reverse was also observed in two patients: low HMG-I(Y) was measured despite high Gleason grade and stage. Nevertheless, HMG-I(Y) expression might be predictive for the malignant potential of prostate cancer cells.

The mechanism by which the HMG-I(Y) gene is regulated in prostate tissue remains to be elucidated. The proteins are present in abundance ($10^5-10^6$ molecules per cell), which indicates that they could be involved in the regulation of many genes, some of which might be involved in cell growth. Recently, Friedmann *et al.* (1993) localised the HMG-I(Y) gene to the short arm of chromosome 6 in a region where rearrangements, translocations and other abnormalities have been found in numbers of human cancers.

HMG-I(Y) has been implicated in a number of functions: the subunits of NF-$\kappa$B (p50 and p65), members of the oncogene *rel* family, can only activate transcription from their binding site PRDII when HMG-I(Y) is also bound (Thanos *et al.*, 1992), similarly the E-selectin gene, encoding for endothelial cell adhesion proteins, can be activated only via interleukin (IL)-13 and tumour necrosis factor (TNF)-$\alpha$ induction of NF-$\kappa$B through HMG-I(Y) binding (Lewis *et al.*, 1994). HMG-I(Y) is involved in rescuing scaffold-associated regions (SARs) and A:T rich sequences from histone H1-mediated repression, which fold the chromatin fibre into higher order structures (Zhao *et al.*, 1993); finally HMG-I(Y) plays a role in the suppression of IL-4 transcription in T lymphocytes (Chuvpilo *et al.*, 1993), as well as in the stimulation of a specific isoform of the activating transcription factor 2 ($ATF\text{-}2_{195}$) binding to interferon $\beta$ (IFN-$\beta$) (Du *et al.*, 1994). In view of the multifunctionality of HMG-I(Y) in mammalian cells, it is not

**Table II** Statistical analysis of data obtained on paraffin tissue from a patient who underwent radical prostatectomy

| *Group* | *Mean values of A* | *± s.d.* | t-*value* | P-value |
|---|---|---|---|---|
| Gg 1–2 | 0.42 | ±0.16 | 4.79 | (<0.0001) |
| Gg 3 | 0.95 | ±0.38 | | |
| Gg 1-2 | 0.42 | ±0.16 | 6.03 | (<0.0001) |
| Gg 4–5 | 1.34 | ±0.52 | | |
| Gg 3 | 0.95 | ±0.38 | 3.07 | (=0.0030) |
| Gg 4–5 | 1.34 | ±0.52 | | |

*t*-test was performed to compare (two by two) the groups for their HMG-I(Y) expression. Gg, Gleason grade.

**Table III** Relation between HMG-I(Y) expression levels and stages

| | *T1* | *T2* | *T3* | *T4* |
|---|---|---|---|---|
| A<0.65 | 0 | 13 | 4 | 0 |
| A>0.65 | 1 | 13 | 40 | 1 |

Correlation between stage and HMG-I(Y) expression over and below a threshold of 0.65 (normalised score A).

**Figure 2** Correlation of HMG-I(Y) expression with Gleason grade (Gg) in prostate cancer.

**Figure 3** Kaplan-Meier progression free rate related to HMG-I(Y) expression. Log-rank test: chi-square = 5.017, $P=0.025$. (——) HMG-I(Y)<0.65; (- - - -), HMG-I(Y)>0.65.

surprising that high HMG-I(Y) expression is important in cancer progression. The results presented here indicate that measurement of HMG-I(Y) levels in prostate cancer may be useful as a prognostic marker. However, the technical difficulties of RISH should be taken into account for routine use. The development of less cumbersome techniques for HMG-I(Y) detection, i.e. immunohistology, is necessary before this can be implemented.

**Acknowledgements**
The authors gratefully acknowledge Dr Pierre P Bringuier, Dr Marion JG Bussemakers and Dr Egbert Oosterwijk for critically reading the manuscript. We thank Dr van Iersel for providing patient data, Mrs Tilly W Aalders, Mr A van Bokhoven, and Mr Peter van Stratum for their excellent assistance and help. This work was supported by the Dutch Cancer Foundation NUKC 9001 and FUSEX (YT).

### References

ADOLFSSON J, RONSTROM L, CARSTENSEN J, LOWHAGEN T AND HEDLUND PO. (1990). The natural course of low grade, non-metastatic prostatic carcinoma. *Br. J. Urol.*, **65,** 611–614.

BORING CC, SQUIRES TS, TONG T AND MONTGOMERY S. (1994). Cancer statistics, 1994. *CA. Cancer J. Clin.*, **44,** 7–26.

BOSTWICK DG, GRAHAM SD JR, NAPALKOV P, ABRAHAMSSON PA, DI SANT'AGNESE PA AND ALGABA F. (1993). Staging of early prostate cancer: a proposed tumour volume-based prognostic index. *Urology*, **41,** 403–411.

BOSTWICK DG, MONTIRONI R, NOGLE R, PRETLOW T, MILLER G AND WHEELER T. (1992). Current and proposed biologic markers in prostate cancer. *J. Cell. Biochem.*, **16H** (Suppl.), 65–67.

BUSSEMAKERS MJ, VAN DE VEN WJ, DEBRUYNE FM AND SCHALKEN JA. (1991). Identification of high mobility group protein I(Y) as potential progression marker for prostate cancer by differential hybridization analysis. *Cancer Res.*, **51,** 606–611.

CHUVPILO S, SCHOMBERG C, GERWIG R, HEINFLING A, REEVES R AND GRUMMT F. (1993). Multiple closely-linked NFAT/octamer and HMG I(Y) binding sites are part of the interleukin-4 promoter. *Nucleic Acids Res.*, **21,** 5694–5704.

DISNEY JE, JOHNSON KR, MAGNUSON NS, SYLVESTER SR AND REEVES R. (1989). High-mobility group protein HMG-I localizes to G/Q- and C-bands of human and mouse chromosomes. *J. Cell Biol.*, **109,** 1975–1982.

DU W AND MANIATIS T. (1994). The high mobility group protein HMG I(Y) can stimulate or inhibit DNA binding of distinct transcription factor ATF-2 isoforms. *Proc. Natl Acad. Sci. USA*, **91,** 11318–11322.

ELTON TS AND REEVES R. (1986). Purification and postsynthetic modifications of Friend erythroleukemic cell high mobility group protein HMG-I. *Anal. Biochem.*, **157,** 53–62.

FASHENA SJ, REEVES R AND RUDDLE NH. (1992). A poly(dA-dT) upstream activating sequence binds high-mobility group I protein and contributes to lymphotoxin (tumour necrosis factor-beta) gene regulation. *Mol. Cell. Biol.*, **12,** 894–903.

FRIEDMANN M, HOLTH LT, ZOGHBI HY AND REEVES R. (1993). Organization, inducible-expression and chromosome localization of the human HMG-I(Y) nonhistone protein gene. *Nucleic Acids Res.*, **21,** 4259–4267.

GIANCOTTI V, BURATTI E, PERISSIN L, ZORZET S, BALMAIN A AND PORTELLA G. (1989). Analysis of the HMGI nuclear proteins in mouse neoplastic cells induced by different procedures. *Exp. Cell Res.*, **184,** 538–545.

GIANCOTTI V, PANI B, D'ANDREA P, BERLINGIERI MT, DI FIORE PP AND FUSCO A. (1987). Elevated levels of a specific class of nuclear phosphoproteins in cells transformed with v-*ras* and v-*mos* oncogenes and by cotransfection with c-*myc* and polyoma middle T genes. *EMBO J.*, **6,** 1981–1987.

GLEASON DF. (1977). Histological grading and clinical staging of prostate carcinoma. In *Urologic Pathology: the Prostate*, M Tannenbaum (ed.), pp. 171–197. Lea and Febiger: Philadelphia.

HAYASHI S, GILLAM IC, DELANEY AD AND TENER GM. (1978). Acetylation of chromosome squashes of *Drosophila melanogaster* decreases the background in autoradiographs from hybridization with [$^{125}$I]-labeled RNA. *J. Histochem. Cytochem.*, **26,** 677–679.

JOHANSSON JE, ADAMI HO, ANDERSSON SO, BERGSTROM R, KRUSEMO UB AND KRAAZ W. (1989). Natural history of localised prostatic cancer. A population-based study in 223 untreated patients. *Lancet*, **1,** 799–803.

JOHNSON KR, LEHN DA, ELTON TS, BARR PJ AND REEVES R. (1988). Complete murine cDNA sequence, genomic structure, and tissue expression of the high mobility group protein HMG-I(Y). *J. Biol. Chem.*, **263,** 18338–18342.

JOHNSON KR, LEHN DA AND REEVES R. (1989). Alternative processing of mRNAs encoding mammalian chromosomal high-mobility-group proteins HMG-I and HMG-Y. *Mol. Cell. Biol.*, **9,** 2114–2123.

KAPLAN EL AND MEIER P. (1958). Non parametric estimation from incomplete observations. *J. Am. Stat. Assoc.*, **53,** 457–481.

LEWIS H, KASZUBSKA W, DELAMARTER JF AND WHELAN J. (1994). Cooperativity between two NF-kappa B complexes, mediated by high mobility-group protein I(Y), is essential for cytokine-induced expression of the E-selectin promoter. *Mol. Cell. Biol.*, **14,** 5701–5709.

LUND T, HOLTLUND J, FREDRIKSEN M AND LALAND SG. (1983). On the presence of two new high mobility group-like proteins in HeLa S3 cells. *FEBS Lett.*, **152,** 163–167.

MANFIOLETTI G, GIANCOTTI V, BANDIERA A, BURATTI E, SAUTIERE P AND CARY P. (1991). cDNA cloning of the HMGI-C phosphoprotein, a nuclear protein associated with neoplastic and undifferentiated phenotypes. *Nucleic Acids Res.*, **19,** 6793–6797.

MEIJER L, OSTVOLD AC, WALASS SI, LUND T AND LALAND SG. (1991). High-mobility-group proteins P1, I and Y as substrates of the M-phase-specific p34cdc2/cyclincdc13 kinase. *Eur. J. Biochem.*, **196,** 557–567.

NAGLE RB, BRAWER MK, KITTELSON J AND CLARK V. (1991). Phenotypic relationships of prostatic intraepithelial neoplasia to invasive prostatic carcinoma. *Am. J. Pathol.*, **138,** 119–128.

RAM TG, REEVES R AND HOSICK HL. (1993). Elevated high mobility group-I(Y) gene expression is associated with progressive transformation of mouse mammary epithelial cells. *Cancer Res.*, **53,** 2655–2660.

SKALNIK DG AND NEUFELD EJ. (1993). Sequence-specific binding of HMG-I(Y) to the proximal promoter of the gp91-phox gene. *Biochem. Biophys. Res. Commun.*, **190,** 308–309.

SKALOVA A, LEHTONEN H, VON BOGUSLAWSKY K AND LEIVO I. (1994). Prognostic significance of cell proliferation in mucoepidermoid carcinomas of the salivary gland: clinicopathological study using MIB 1 antibody in paraffin sections. *Hum. Pathol.*, **25,** 929–935.

SOLOMON MJ, STRAUSS F AND VARSHAVSKY A. (1986). A mammalian high mobility group protein recognizes any stretch of six A.T base pairs in duplex DNA. *Proc. Natl Acad. Sci. USA*, **83,** 1276–1280.

TAMIMI Y, VAN DER POEL HG, DENYN MM, UMBAS R, KARTHAUS HF, DEBRUYNE FM AND SCHALKEN JA. (1993). Increased expression of high mobility group protein I(Y) in high grade prostatic cancer determined by in situ hybridization. *Cancer Res.*, **53,** 5512–5516.

THANOS D AND MANIATIS T. (1992). The high mobility group protein HMG I(Y) is required for NF-kappa B-dependent virus induction of the human IFN-beta gene. *Cell*, **71,** 777–789.

UMBAS R, ISAACS WB, BRINGUIER PP, SCHAAFSMA HE, KARTHAUS HF, OOSTERHOF GO, DEBRUYNE FM AND SCHALKEN JA. (1994). Decreased E-cadherin expression is associated with poor prognosis in patients with prostate cancer. *Cancer Res.*, **54,** 3929–3933.

UMBAS R, SCHALKEN JA, AALDERS TW, CARTER BS, KARTHAUS HF, SCHAAFSMA HE, DEBRUYNE FM AND ISAACS WB. (1992). Expression of the cellular adhesion molecule E-cadherin is reduced or absent in high-grade prostate cancer. *Cancer Res.*, **52,** 5104–5109.

VARTIAINEN E, PALVIMO J, MAHONEN A, LINNALA KANKKUNEN A AND MAENPAA PH. (1988). Selective decrease in low-Mr HMG proteins HMG I and HMG Y during differentiation of mouse teratocarcinoma cells (published erratum appears in *FEBS Lett.* 1989 Jul 17;251(1-2):283). *FEBS Lett.*, **228**, 45-48.

WEGNER M AND GRUMMT F. (1990). Netropsin, distamycin and berenil interact differentially with a high-affinity binding site for the high mobility group protein HMG-I. *Biochem. Biophys. Res. Commun.*, **166**, 1110-1117.

YANG YEN HF AND ROTHBLUM LI. (1988). Purification and characterization of a high-mobility-group-like DNA-binding protein that stimulates rRNA synthesis in vitro. *Mol. Cell. Biol.*, **8**, 3406-3414.

ZHAO K, KAS E, GONZALEZ E AND LAEMMLI UK. (1993). SAR-dependent mobilization of histone H1 by HMG-I/Y in vitro: HMG-I/Y is enriched in H1-depleted chromatin. *EMBO J.*, **12**, 3237-3247.